# Complete mitogenome of Intermediate Egret *Ardea intermedia* (Ciconiiformes: Ardeidae)

**DOI:** 10.1080/23802359.2017.1361352

**Published:** 2017-08-06

**Authors:** Feiyun Tu, Shan Tang, Chaochao Yan, Xiaofeng Huang

**Affiliations:** a Institute of Wildlife Conservation, Jiangxi Academy of Forestry, Nanchang, Jiangxi Province, China;; b Sichuan Key Laboratory of Conservation Biology on Endangered Wildlife, College of Life Sciences, Sichuan University, Chengdu, Sichuan Province, China

**Keywords:** Ardeidae, *Ardea intermedia*, complete mitogenome, conservation genetics

## Abstract

The Intermediate Egret *Ardea intermedia* belongs to family Ardeidae, and it is widely distributed over east Africa across the Indian subcontinent to Southeast Asia and Australia. In the present study, the total mitochondrial genome of *A. intermedia* was determined. The genome is 18,578 bases in length and contains 13 protein-coding genes (PCGs), 22 transfer RNA genes, 2 ribosomal RNA genes and 2 non-coding regions (CR and CRR), with a base composition of A 30.8%, G 14.0%, T 24.3% and C 30.9%. The dn/ds values of ten PCGs (ND1, ND2, ATP8, ATP6, Cox3, ND3, ND4L, ND4, ND5 and ND6) are below 1. Bayesian inference (BI) and maximum likelihood (ML) methods generated similar topologies. Phylogenies showed that *Ardea novaehollandiae* and *A. intermedia* should be assign to *Egretta* and *Ardea*, respectively. The mitogenomic data of *A. intermedia* will be useful in the conservation genetics and phylogeny of the species.

The Intermediate Egret *Ardea intermedia* is a medium-sized white egret in the family Ardeidae. It is a resident breeder from east Africa across the Indian subcontinent to Southeast Asia and Australia. The wild population is decreasing and the species has been evaluated as Least Concern by IUCN (IUCN 2017). So far, limited molecular data on the species have been reported (Jansen et al. 2009; Zhou et al. 2014). Here, we sequenced and characterized the complete mitogenome of *A. intermedia*, with the individual (JAF001) captured in Yongxiu (115.888^o^E, 29.067^o^N), Jiangxi Province and which was preserved in Jiangxi Academy of Forestry, to provide basis data for conservation genetics and evolution of this species.

Total genomic DNA was extracted from tissue samples using standard phenol/chloroform methods (Sambrook and Russell 2001). The PCR protocol followed the study of Zhou et al. (2014) and made several modifications.

In the present study, the complete mitogenome of *A. intermedia* (GenBank Accession Number KX592585, 18,578 bp), is comprised of 13 protein-coding genes (PCGs), 22 transfer RNA genes, 2 ribosomal RNA genes and 2 non-coding regions (CR and CCR) (Table S1). The gene order of *A. intermedia* was identical to that observed in the most birds (Zhou et al. 2014; Huang et al. 2016, 2017). The total base composition of the mitochondrial genome is showed as follows: A 30.8%, G 14.0%, T 24.3% and C 30.9%, with an A + T-rich pattern (Zhou et al. 2014).

To check the selection pressure within two individuals of the species, we calculated dn/ds based on the 13 PCGs with corresponding sequences available in GenBank (NC_025918) as implemented by FasParser v1.2.1 (Sun 2017). The dn/ds values of most PCGs (ND1, ND2, ATP8, ATP6, Cox3, ND3, ND4L, ND4, ND5 and ND6) are below 1, to the exclusion of Cox1, Cox2 and Cytb.

We also retrieved 13 PCGs of 26 species. Bayesian inference (BI) and maximum likelihood (ML) methods were performed based on concatenated 13 PCGs. BI analysis was carried out as mentioned by Tu et al. (2012). ML was performed using PhyML 3.0 online platform (Guindon et al. 2010) with default parameters. Phylogenies based on BI and ML showed similar topologies (Figure 1). The phylogeny ((Ardeidae, Threskiornithidae) Ciconiidae) was highly supported with the BI analysis. *A. novaehollandiae* clustered with Clade B rather than Clade A, and thus *A. novaehollandiae* should be assigned to the genus *Egretta*. The results were consistent with previous studies (Chen et al. 2000; Zheng 2011). *Ardea modesta* was sister relationship to *A. intermedia*.

**Figure 1.**
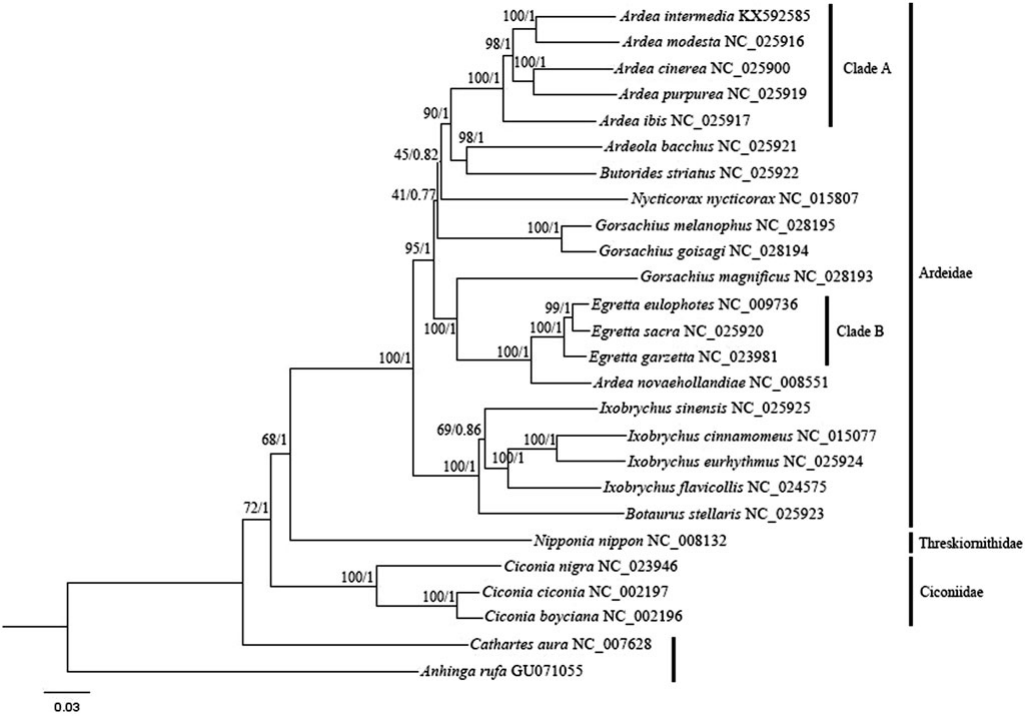
Bayesian 50% majority-rule consensus phylogenetic tree of 26 species based on 13 PCGs. *Cathartes aura* and *Anhinga rufa* were selected as out-groups. The numbers on the internode branches are Bayesian posterior probability and bootstrap percentages for the ML analysis.

Until now, the generic placement of *A. intermedia* has been disputed. The species was placed in the genus *Mesophoyx* (MacKinnon et al. 2000), while some authorities (Chen et al. 2000; Zheng 2011) listed it in genus *Egretta*. In this study, the species should be considered in the genus *Ardea* and the results were in concordant with Zhou et al. (2014). Complete mitochondrial genome sequences within the family Ardeidae are still deficient and the molecular data may contribute to the conservation genetics and the phylogenetic evolution position of the species.

## Supplementary Material

TMDN_A_1361352_Supplementary_Information.zip
