# Supplementary material for: Complete mitogenome of Intermediate Egret Ardea intermedia (Ciconiiformes: Ardeidae)
Source: Mitochondrial DNA B Resour. 2017 Aug 6;2(2):510–1. doi: 10.1080/23802359.2017.1361352 (PMC7799489; doi:10.1080/23802359.2017.1361352)
Supplement: TMDN_A_1361352_Supplementary_Information.zip [file TMDN_A_1361352_SM5041.zip › Table S1.doc]

*Table S1 Characteristics of mitochondrial genome for Ardea intermedia*

|  | **Position** | |  |  | **Codon** | |
| --- | --- | --- | --- | --- | --- | --- |
| **Gene** | **From** | **To** | **Size (bp)** | **Strand** | **Start** | **Stop** |
| tRNA-Phe | 1 | 68 | 68 | H |  |  |
| 12S rRNA | 69 | 1039 | 971 | H |  |  |
| tRNA-Val | 1040 | 1111 | 72 | H |  |  |
| 16S rRNA | 1112 | 2704 | 1593 | H |  |  |
| tRNA-Leu(UUR) | 2705 | 2778 | 74 | H |  |  |
| *ND1* | 2789 | 3766 | 978 | H | ATG | AGA |
| tRNA-Ile | 3773 | 3843 | 71 | H |  |  |
| tRNA-Gln | 3854 | 3924 | 71 | L |  |  |
| tRNA-Met | 3924 | 3992 | 69 | H |  |  |
| *ND2* | 3993 | 5033 | 1041 | H | ATG | TAG |
| tRNA-Trp | 5032 | 5103 | 72 | H |  |  |
| tRNA-Ala | 5105 | 5173 | 69 | L |  |  |
| tRNA-Asn | 5185 | 5258 | 74 | L |  |  |
| tRNA-Cys | 5264 | 5330 | 67 | L |  |  |
| tRNA-Tyr | 5330 | 5401 | 72 | L |  |  |
| *Cox1* | 5403 | 6953 | 1551 | H | GTG | AGG |
| tRNA-Ser(UCN) | 6945 | 7018 | 74 | L |  |  |
| tRNA-Asp | 7021 | 7089 | 69 | H |  |  |
| *Cox2* | 7091 | 7774 | 684 | H | ATG | TAA |
| tRNA-Lys | 7776 | 7846 | 71 | H |  |  |
| *ATP8* | 7848 | 8015 | 168 | H | ATG | TAA |
| *ATP6* | 8006 | 8689 | 684 | H | ATG | TAA |
| *Cox3* | 8689 | 9472 | 784 | H | ATG | T |
| tRNA-Gly | 9473 | 9541 | 69 | H |  |  |
| *Nd3* | 9542 | 9893 | 352 | H | ATT | TAA |
| tRNA-Arg | 9896 | 9964 | 69 | H |  |  |
| *ND4L* | 9966 | 10262 | 297 | H | ATG | TAA |
| *ND4* | 10256 | 11633 | 1378 | H | ATG | T |
| tRNA-His | 11634 | 11703 | 70 | H |  |  |
| tRNA-Ser(AGY) | 11704 | 11769 | 66 | H |  |  |
| tRNA-Leu(CUN) | 11769 | 11838 | 70 | H |  |  |
| *ND5* | 11839 | 13653 | 1815 | H | ATG | AGA |
| *Cytb* | 13665 | 14807 | 1143 | H | ATG | TAA |
| tRNA-Thr | 14811 | 14880 | 70 | H |  |  |
| tRNA-Pro | 14899 | 14972 | 74 | L |  |  |
| CR | 14973 | 16502 11116502 | 1530 | H |  |  |
| *ND6* | 16503 | 17024 | 522 | L | ATG | AGG |
| tRNA-Glu | 17028 | 17101 | 74 | L |  |  |
| CCR | 17102 | 18578 | 1477 | H |  |  |
